# Nomogram predicting the risk of three-year chronic kidney disease adverse outcomes among East Asian patients with CKD

**DOI:** 10.1186/s12882-021-02496-7

**Published:** 2021-09-27

**Authors:** Huizhen Ye, Youyuan Chen, Peiyi Ye, Yu Zhang, Xiaoyi Liu, Guanqing Xiao, Zhe Zhang, Yaozhong Kong, Gehao Liang

**Affiliations:** 1 Nephrology Department, The First People’s Foshan Hospital, Foshan, Guangdong China; 2 Staff Health Care Department, Sun Yat-sen University Cancer Center, State Key Laboratory of Oncology in South China, Collaborative Innovation Center for Cancer Medicine, Guangzhou, China; 3 Department of Breast Oncology, Sun Yat-sen University Cancer Center, State Key Laboratory of Oncology in South China, Collaborative Innovation Center for Cancer Medicine, Guangzhou, China

**Keywords:** Nomogram, East Asian patients with CKD, 3-year adverse-outcome-free probability, CKD progression

## Abstract

**Background:**

Chronic kidney disease (CKD) is a common health challenge. There are some risk models predicting CKD adverse outcomes, but seldom focus on the Mongoloid population in East Asian. So, we developed a simple but intuitive nomogram model to predict 3-year CKD adverse outcomes for East Asian patients with CKD.

**Methods:**

The development and internal validation of prediction models used data from the CKD-ROUTE study in Japan, while the external validation set used data collected at the First People’s Hospital of Foshan in southern China from January 2013 to December 2018. Models were developed using the cox proportional hazards model and nomogram with SPSS and R software. Finally, the model discrimination, calibration and clinical value were tested by R software.

**Results:**

The development and internal validation data-sets included 797 patients (191 with progression [23.96%]) and 341 patients (89 with progression [26.10%]), respectively, while 297 patients (108 with progression [36.36%]) were included in the external validation data set. The nomogram model was developed with age, eGFR, haemoglobin, blood albumin and dipstick proteinuria to predict three-year adverse-outcome-free probability. The C-statistics of this nomogram were 0.90(95% CI, 0.89–0.92) for the development data set, 0.91(95% CI, 0.89–0.94) for the internal validation data set and 0.83(95% CI, 0.78–0.88) for the external validation data-set. The calibration and decision curve analyses were good in this model.

**Conclusion:**

This visualized predictive nomogram model could accurately predict CKD three-year adverse outcomes for East Asian patients with CKD, providing an easy-to-use and widely applicable tool for clinical practitioners.

**Supplementary Information:**

The online version contains supplementary material available at 10.1186/s12882-021-02496-7.

## Background

Chronic kidney disease (CKD) is becoming a common global health challenge. A report in 2016 pointed out that the prevalence of all stages of CKD varies between 7 and 12% in the different regions of the world [1]. In 2019, a 15% overall prevalence of CKD was reported in adults in the United States [2]. Patients with CKD have an increased risk of deteriorating into end-stage renal disease (ESRD), requiring dialysis to survive or suffering from complications such as cardiovascular events or even death. With CKD progression and a high risk of other adverse outcomes, the costs for healthcare and therapies are increasing [3]. Accurate risk prediction for CKD adverse outcomes could contribute to individualized decision making, early patient therapy performance, complication reduction and dialysis preparation [4].

There are some risk models predicting the progression and some adverse outcomes of CKD [5]. Many different risk factors were applied in previous models, and the area under the curve (AUC) of these models ranged from 0.56 to 0.94, with calibration from modest to good [6]. However, there were some limitations for the models mentioned above. First, few of these models have been externally validated. Risk model validation includes internal validation (validating in the same population as the model was developed) and external validation (validating in another population). When the AUC performance was generally acceptable-to-good on the derivation population as internal validation, the AUCs were usually modest-to-acceptable in a new population, even though some AUCs for external validation were not reported in most studies. Second, most of the models focus on only one kind of CKD [7, 8], with many risk factors or even some novel professional biomarkers [9], which are complicated and difficult to widely use, especially in grassroots hospitals or community hospitals. Third, the previous models were mainly derived from white populations, but seldom paid attention to East Asian patients with CKD. It was pointed out that the progression and some adverse outcomes of CKD also varied within countries by ethnicity [10]. Black and Asian people in the UK, Hispanics in the USA, and indigenous people in some other developed countries are at higher risk of CKD progression [11]. Given these inconvenience, it is necessary to validate a simple, intuitive and easily applied predictive model to predict adverse outcomes of CKD for the Mongoloid CKD population in East Asia.

Nomograms are a kind of predictive tools, representing statistical predictive model by a number of scales, and generate the probability of a clinical event at appropriate values [12]. Using data from two different populations, we aimed to develop a simple but intuitive model to predict CKD progression for the East Asian CKD population that can be easily and widely implemented for clinical practitioners. To make our findings more credible, the public data were randomly split at a ratio of 7:3, into a developing and another internal validation data set. The enrolled data from our hospital were analysed for external validation. Model validation including discrimination, calibration and clinical values, was assessed in all three datasets.

## Methods

### Study population

#### Development data set

The development cohort was derived from the Chronic Kidney Disease Research of Outcomes in Treatment and Epidemiology (CKD-ROUTE) study [13]. This was a prospective, observational cohort study in Japan. Written informed consent was obtained from all patients, which was mentioned in their paper [13]. The CKD-ROUTE study was approved by the ethics committees of Tokyo Medical and Dental University, School of Medicine. All the population was in stage G2–G5 CKD according to the Kidney Disease Improving Global Outcomes (KDIGO) classification and was not undergoing dialysis [14]. Patients who were newly visiting nephrology centres from October 2010 to December 2011 and older than 20 years old were included. Subjects were excluded if they had malignancy, transplantation, active gastrointestinal bleeding or no written informed consent. Over 1000 participants were recruited at the Tokyo Medical and Dental University Hospital and its 15 affiliated hospitals. Participants visited the hospital every 6 months for assessment of their clinical status. The observation duration was 22.91 ± 14.60 months with a range from 1 to 39 months. All the CKD-ROUTE data were from the Dryad data package [15] of its original publication [13] from the Dryad Digital Repository, a public resource that provides discoverable, freely reusable, and citable data. For this analysis, no informed consent was required from CKD-ROUTE patients since all the data were deidentified, and the Ethics Committee of the First Hospital of Foshan in China approved the study with Number 64 in 2020.

#### Validation data set

The validation consisted of internal validity and external validity. The validation cohort was also derived from the CKD-ROUTE study. The external validation was from retrospective data collected at the First Hospital of Foshan in China from January 2013 to December 2018. Patients with CKD stage G2-G5 hospitalized in Foshan Hospital were included, but those with malignancy, transplantation, active gastrointestinal bleeding and without follow-up visits in our hospital were excluded. Finally, 297 patients in total were recruited. All patients in Foshan Hospital in China also provided written informed consents.

### Variables

At the time of enrolment, candidate dependent variables were selected by literature research, previous studies [6] and the data we attained in the CKD-ROUTE study, including age, sex, body mass index(BMI), aetiology of CKD, blood pressure, albumin, haemoglobin, eGFR, dipstick proteinuria, case history, CKD stages and urinary occult blood. Biochemical variables were collected by testing blood and urine samples. The eGFR in all populations was calculated from the formula: eGFR = 194 × serum creatinine − 1.094 × age − 0.287 (if female, × 0.739). It was calculated using the modified three-variable Modification of Diet in Renal Disease equation developed by the Japanese Society of Nephrology [16]. CKD was classified according to the Kidney Disease Improving Global Outcomes(KDIGO) guideline [14], which is defined as G2, G3, G4 and G5 if the corresponding eGFR (mL/min/1.73 m2) is 60–89, 45–59, 30–44,15–29 and < 15. In addition, none of the patients were undergoing dialysis. Dipstick proteinuria was defined as − 1 or 0 as negative or trace protein by dipstick urinary test at enrolment. Dipstick proteinuria 1 to 4 represented the degree of proteinuria from 1 to 4.

Consistent with the CKD-ROUTE study, the primary endpoint was CKD adverse outcomes, which were defined as > 50% eGFR loss, initiation of dialysis in ESRD, cardiovascular events (CVEs), and all-cause death. CVEs included ischaemic heart disease, congestive heart failure, peripheral arterial disease, or stroke.

### Sample size

Usually, the effective sample size is often associated with the number of outcome events. According to previous rules and experience, at least 10 events should be ensured per candidate predicted variable before variable selection [17–19].

### Statistical analysis

The CKD-ROUTE dataset was randomly divided into two cohorts with the R software (i386 3.5.3)– development dataset (70% of the total data) and internal validation dataset (30% of the total data). The dataset from the First Hospital of Foshan was used for the external validity. Baseline continuous characteristics of all datasets are presented as the means ± standard deviation and were compared by paired Student’s t-test if the data were normally distributed or by the paired rank-sum test for non-normally distributed data. Other categorical data between two groups are expressed as numbers and percentages and were compared by using the paired chi square test. All probabilities were two-tailed and the level of significance was 0.05.

To develop the model, first, we tested the associations between potential variables and CKD adverse outcomes by univariable and multivariable Cox proportional hazards models with SPSS version 22.0 (Chicago, IL, USA). *P* values of < 0.05 were considered statistically significant. Next, a predictive nomogram was developed with variables selected from the Cox analysis with R software.

### Model validation

The validation of our model was tested with different methods in different aspects.

First, the discriminations, the ability of the model to separate individuals who develop events from those who do not, was evaluated by the C-statistic in all three data sets, which is defined as perfect, good, moderate and poor if the corresponding figure is 1, > 0.8, 0.6–0.8 and < 0.6, respectively [20]. The C-statistics for the predictor eGFR were also calculated in these three data sets.

Second, the calibration (or goodness-of-fit) [21] was tested by calibration plots. The calibration was good if the calibration line between the predicted probability and the observed outcome fitted to the ideal standard line(y = x).

Third, decision curve analysis (DCA) was used to test the clinical value of our model and visualize the potential net benefit of the model [22]. The model DCA was compared with the DCA of predictor eGFR and other variables.

All statistical analyses above were performed using R software. All probabilities were two-tailed and the level of significance was 0.05.

## Results

The characteristics of patients at baseline in the development and validation data sets are listed in Table 1. The development and internal validation data set included 797 patients (191 with adverse outcomes [23.96%]) and 341 patients (89 with adverse outcomes [26.10%]) from CKD-ROUTE data set. The *P* value of sex, body mass index(BMI), aetiology of CKD, blood pressure(mmHg), serum albumin(g/dL), haemoglobin(g/dL), eGFR (ml/min/1.73 m^2^), case history, dipstick proteinuria, urinary occult blood, medication usage and adverse outcomes were all > 0.05, showing no significant difference between these two groups. On the other hand, 297 patients (108 with progression [36.36%]) from the First Hospital of Foshan were included in the external validation data set. The variables between development data set and external validation data-set were significantly different with *P* < 0.05, demonstrating that these two datasets completely varied.

**Table 1:** Characteristics of patients at baseline in the training and validation data sets

Variables	Development data set	Internal validity data set	*P*-value	External validity data set	*P*-value
Age (years)	67.50 ± 13.52 (66.5–68.5)	67.82 ± 13.60 (66.22–69.4)	0.95	51.77 ± 16.50 (49.70–53.84)	< 0.01
Sex, n(%)			0.31		< 0.01
Male	570 (72.06%)	222 (65.10%)		158 (53.20%)	
Female	227 (27.94%)	119 (34.90%)		139 (47.80%)	
BMI (kg/m2)	23.71 ± 4.04 (23.41–24.01)	23.80 ± 4.10 (23.32–24.28)	0.78	23.36 ± 3.87 (23.36–24.33)	0.59
Aetiology of CKD			0.73		< 0.01
Diabetic nephropathy, n(%)	194 (24.34%)	93 (27.27%)		40 (13.47%)	
Nephrosclerosis, n (%)	325 (40.78%)	126 (36.95%)		17 (5.72%)	
Glomerulonephritis, n (%)	150 (18.82%)	66 (19.36%)		177 (59.60%)	
Other, n (%)	128 (16.06%)	56 (16.42%)		63 (21.21%)	
Systolic blood pressure (mmHg)	139.64 ± 22.52 (137.98–141.31)	141.16 ± 22.83 (138.48–143.83)	0.57	140.16 ± 25.86 (136.92–143.41)	0.23
Serum albumin(g/dL)	3.85 ± 0.62 (3.80–3.89)	3.80 ± 0.71 (3.72–3.89)	0.75	3.64 ± 0.69 (3.56–3.73)	< 0.01
Haemoglobin(g/dL)	11.89 ± 2.34 (11.72–12.06)	11.83 ± 2.09 (11.59–12.08)	0.89	11.48 ± 2.53 (11.16–11.80)	< 0.01
eGFR (ml/min/1.73 m^2^)	32.46 ± 18.47 (31.09–33.83)	31.09 ± 18.54 (28.92–33.26)	0.34	38.80 ± 23.92 (35.79–41.80)	0.01
Hypertension, n(%)	719 (90.21%)	308 (90.32%)	0.96	178 (59.93%)	0.01
Cardiovascular disease, n(%)	220 (27.60%)	85 (24.93%)	0.35	56 (18.86%)	
Diabetic, n(%)	285 (35.76%)	137 (40.18%)	0.16	70 (23.57%)	
Dipstick proteinuria, n (%)			0.16		< 0.01
-1	183 (23.28%)	75 (22.32%)		39 (13.13%)	
0	109 (13.87%)	45 (13.39%)		12 (4.04%)	
1	112 (14.25%)	51 (15.58%)		45 (15.15%)	
2	173 (22.01%)	73 (21.73%)		81 (27.27%)	
3 or 4	209 (26.58%)	92 (27.38%)		120 (40.41%)	
CKD stages			0.43		< 0.01
1	0	0		10 (3.37%)	
2	63 (7.90%)	32 (9.38%)		48 (16.16%)	
3	337 (42.28%)	133 (39%)		106 (35.69%)	
4	261 (32.75%)	103 (30.21%)		88 (29.63%)	
5	136 (17.06%)	73 (21.41%)		45 (15.15%)	
Urinary occult blood(%)	263 (33.46%)	115 (34.23%)	0.8	183 (61.62%)	< 0.01
Medication usage					
RAS inhibitors	499 (37.39%)	221 (35.19%)	0.48	215 (72.39%)	< 0.01
CCB	379 (47.55%)	157 (46.04%)	0.64	202 (68.01%)	< 0.01
Diuretics	261 (32.74%)	120 (35.19%)	0.42	198 (66.67%)	< 0.01
Adverse outcomes (%)			0.44		< 0.01
No	606 (76.04%)	252 (73.9%)		189 (63.64%)	
Yes	191 (23.96%)	89 (26.10%)		108 (36.36%)	
eGFR halving	/	/		21 (7.07%)	
ESRD	/	/		75 (25.25%)	
CVEs	/	/		9 (3.03%)	
death	/	/		3 (1.01%)	
chronic disease					
hepatic disease	/	/		23 (7.74%)	
cancers	/	/		13 (4.38%)	

Values are mean ± [SD](95% CI confidence interval); Values are number(percentage); *BMI* Body Mass Index, *CVEs* cardiovascular events, *ESRD* end-stage renal disease, *CCB* Calcium Channel Blockers

A total of 12 predictive variables before variable selection should ensured that at least 120 events occur. In our development data set, a total of 797 patients and CKD adverse outcomes occurred in 191(23.96%) patients, showing a sample size larger than 120 events, which means that the sample of our predictive model was adequate.

**Fig. 1:**
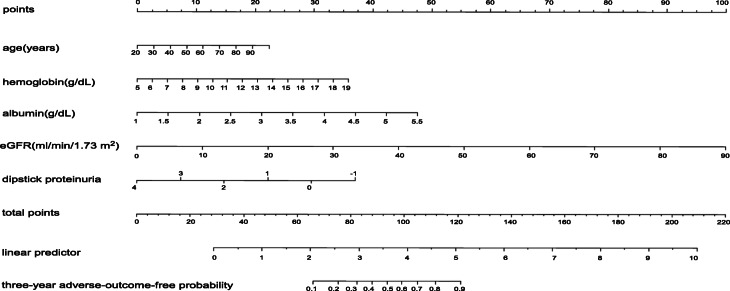
Nomogram of predictors for predicting three-year adverse-outcome-free probability

The variables determined from the Cox proportional hazards model for the nomogram predictive model included age, eGFR, haemoglobin, albumin, and dipstick proteinuria after selection by univariable and multivariable Cox proportional hazards models, with *P* < 0.05(Table 2). The graphical nomogram predicting chronic kidney disease adverse outcomes in 3 years is shown in Fig. 1. The line named three-year adverse-outcome-free probability meant that the patients did not experience adverse outcomes, including > 50% eGFR loss, initiation of dialysis in ESRD, cardiovascular events (CVEs) and all-cause death in 3 years. The C-statistics of this nomogram were 0.90 (95% CI, 0.89–0.92) for the development data set, 0.91 (95% CI, 0.89–0.94) for the internal validation data-set and 0.83 (95% CI, 0.78–0.88) for the external validity data set, all showing good discrimination for our model.

**Table 2:** Selected variables included in nomogram according to Cox proportional hazards model

	Univariable				Multivariable			
variables	HR	95% CI(upper limit value)	95% CI(lower limit value)	*P* value	HR	95% CI(upper limit value)	95% CI(lower limit value)	*P* value
Age (years)	1.07	0.78	1.46	0.68	0.99	0.97	1.00	0.03
Sex	0.99	0.98	1.00	0.40	0.73	0.50	1.05	0.09
BMI (kg/m2)	1.02	0.99	1.06	0.23	0.97	0.93	1.01	0.10
Eatiology of CKD	0.51	0.43	0.61	0.01	0.81	0.64	1.01	0.06
Serum albumin, g/dL	0.57	0.31	0.44	0.01	0.61	0.45	0.81	0.00
Heamoglobin, g/dL	0.68	0.64	0.73	0.01	0.89	0.80	0.98	0.02
eGFR (ml/min/1.73 m2)	0.91	0.89	0.92	0.01	0.91	0.90	0.93	0.00
Dipstick proteinuria	2.09	1.85	2.37	0.01	1.66	1.40	1.97	0.00
Urinary occult blood	1.91	1.43	2.54	0.01	1.21	0.87	1.69	0.25
Hypertension	5.04	1.87	13.58	0.01	0.93	0.33	2.65	0.89
Cardiovascular disease	1.33	0.98	1.81	0.69	0.84	0.59	1.19	0.33
Diabetes	2.86	2.13	3.82	0.01	1.36	0.88	2.08	0.17

*HR* hazard ratio, *CI* confidence interval, *BMI* Body Mass Index

**Fig. 2:**
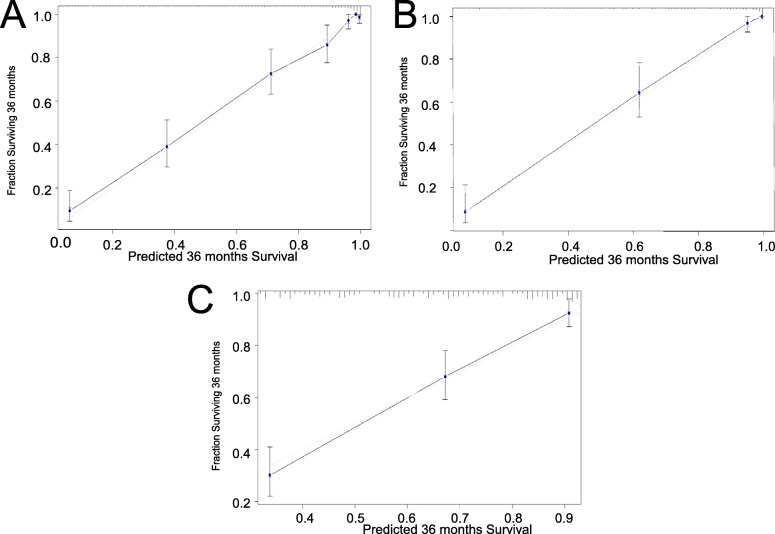
Calibration curves for predicting three-year adverse-outcome-free probability in all data sets. **A**: development data set; **B**: internal validity data set; **C**: external validity data set

The calibration plots for the development, internal validation and external validation datasets are shown in Fig. A, B and C in Fig. 2. They demonstrated good agreement between the predicted probability and the observed outcome, with good fitness to the ideal standard line (light grey line), revealing good calibration of our simple predictive nomogram model.

**Fig. 3:**
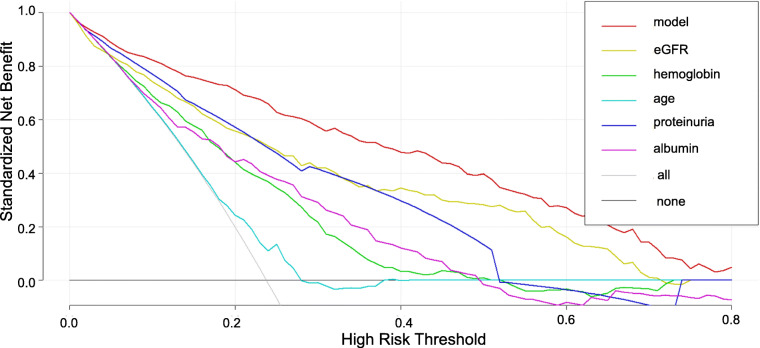
The Decision Curves Analysis curve of nomogram and other factors

The decision curve analysis (DCA) (Fig. 3) curve of our predictive model was farther from the x or y axis than the eGFR curve or other variables alone in our model, which showed better clinical value and potential net benefit in our predictive model.

## Discussion

In our study, the factors associated with CKD adverse outcomes included age, eGFR, haemoglobin, albumin, and dipstick proteinuria. Then, we developed a visualized predictive nomogram model with these five factors to predict 3-year CKD adverse outcomes for the East Asian CKD population. The model validation was good when tested in different aspects and populations, which means this nomogram model was fit for predicting 3-year CKD adverse outcomes and can be easily and widely implemented for clinical practitioners.

In our study, age was found to be a significant risk factor for CKD adverse outcomes, which was different from some previous Asian studies, such as the 2013 [23] and 2019 [24] Taiwan studies. These differences might be due to declining eGFR resulting from loss of muscle mass, reduced physical activity and decreased food intake in elderly patients [25], resulting in age serving as a protective factor in Taiwanese studies. On the other hand, a more common prevalence of CKD was reported in people aged 65 years or older than in people of younger [2]. Furthermore, other CKD risk factors, such as heart disease, obesity, CKD history, and past damage to the kidneys were all more common in the elder.

Proteinuria was also a risk factor for CKD adverse outcomes. In a previous study, proteinuria, a sign of kidney injury, was perceived as the most powerful predictor [26] of kidney damage for promoting inflammation and fibrosis of kidneys [27], which is closely related to a high risk of CKD adverse outcomes. In the MRFIT study, dipstick proteinuria with protein excretion was associated with a greater risk of ESRD [28]. On the other hand, proteinuria was also deemed a strong independent predictor of cardiovascular risk [29], which is the leading cause of death in patients with CKD.

Here, we developed a simple visualized predictive nomogram model to predict CKD 3-year adverse outcomes for East Asian patients and passed internal validation in Japanese patients and external validation in Chinese patients. A 13-year study in Singapore [30] developed a predictive model for CKD in diabetes mellitus, focusing on long-term CKD progression in diabetic nephropathy, which is different from ours. Usually, eGFR is a simple method of monitoring changes in kidney function, estimating CKD adverse outcomes and determining the beginning of dialysis. However, our nomogram showed better discrimination and clinical value than eGFR alone in terms of 3-year adverse-outcome-free probability. In our nomogram, different values of the variables pointed to various corresponding points in line One. For example, consider a 40-year-old patient with an eGFR of 19, haemoglobin 13 g/dl, albumin 3.0 g/dL and 1 dipstick proteinuria; the 40-year age points to approximately 5 points in line 1, while the other factors point to approximately 20 points in line 1, for a total points of 84 points in line 5, which points to a 35% 3-year adverse-outcome-free probability in the last line in Fig. 1. These probabilities could provide physicians and patients with a general scope of prognosis [8] and guide the next clinical steps, such as establishing vascular access for dialysis preparation. Furthermore, a predictive prognosis could offer mental preparation to patients with CKD and help them understand and accept their diseases.

However, our analysis had a few limitations. First, the external validation of our nomogram model was conducted in a retrospective population, but the other data sets were both from a cohort study. Second, the CKD-ROUTE study also reported that factors have certain associations with renal prognosis, but we did not explore them in our model because we did not obtain those data. Further studies are needed. Third, we did not eliminate the possible effect of underlying diseases and medications used for this model. Further studies will be performed and need to be investigated in the future. Fourth, we did not separate the risk of > 50% eGFR loss, initiation of dialysis and other causes of death, but knowing the probability of CKD adverse outcomes is also of benefit to patients and clinical practitioners.

## Conclusion

In conclusion, we developed a visualized predictive nomogram model to predict CKD three-year adverse outcomes for East Asian patients with CKD. The model validation was good when tested in different aspects and populations, offering an easy and widely applicable model for clinical practitioners. However, further prospective population-based studies are needed to investigate the mechanisms.

## Supplementary Information

**Additional file 1.**

**Additional file 2.**

**Additional file 3.**

**Additional file 4.**

**Additional file 5.**

## Data Availability

All data generated or analysed during this study are included in this article and its supplementary information files.

## Abbreviations

CKD: Chronic kidney disease
AUC: Area under the curve
KDIGO: Kidney Disease Improving Global Outcomes
BMI: Body Mass Index
eGFR: Estimated glomerular filtration rate
CVD: Cardiovascular Disease
DCA: Decision curve analyses
CVEs: Cardiovascular events
ESRD: End-stage renal disease
CCB: Calcium Channel Blockers
